# Association of Quantitative Coronary Artery Calcium Density Subtype Volumes With Major Adverse Cardiovascular Events

**DOI:** 10.1016/j.jacadv.2025.102232

**Published:** 2025-10-23

**Authors:** Donghee Han, Aakash Shanbhag, Jianhang Zhou, Sunam Lee, Parker Waechter, Heidi Gransar, Timothy M. Bateman, Robert JH. Miller, John Friedman, Sean Hayes, Louise Thomson, Damini Dey, Daniel S. Berman, Piotr J. Slomka

**Affiliations:** a Departments of Medicine (Division of Artificial Intelligence in Medicine), Imaging and Biomedical Sciences, Cedars-Sinai Medical Center, Los Angeles, California, USA; b Signal and Image Processing Institute, Ming Hsieh Department of Electrical and Computer Engineering, University of Southern California, Los Angeles, California, USA; c Division of Cardiology, Department of Internal Medicine, St. Vincent’s Hospital, The Catholic University of Korea, Seoul, Republic of Korea; d Cardiovascular Imaging Technologies LLC, and Saint Lukes Health System, Kansas City, Missouri, USA; e Department of Cardiac Sciences, University of Calgary, Calgary, Alberta, Canada

**Keywords:** calcium density, calcium volume, computed tomography, coronary artery calcium, major adverse cardiovascular events

## Abstract

**Background:**

Growing evidence has demonstrated that low density coronary artery calcification (CAC) is associated with a higher risk of cardiovascular events.

**Objectives:**

We aim to explore the relationship between CAC volumes at predefined densities, assessed by CAC volume according to CAC Hounsfield unit (HU), and major adverse cardiac events (MACEs).

**Methods:**

We evaluated 3 patient groups with no prior coronary artery disease history who underwent an electrocardiogram-gated noncontrast computed tomography scan for CAC scanning (CAC group, n = 2,028) or as part of a cardiac imaging test: single-photon emission computed tomography (SPECT)-myocardial perfusion imaging (SPECT group, n = 2,782), and positron emission tomography (PET)-myocardial perfusion imaging (PET group, n = 2,366). CAC subtype volumes of low, intermediate, and high density based on HU cutoff (low: 130-199 HU, intermediate: 200-399 HU, and high: ≥400 HU). MACE included mortality, myocardial infarction, unstable angina, and late revascularization.

**Results:**

During a median 4.3 years (interquartile ranges: 2.6-12.8) follow-up duration, 1,033 MACE occurred (14.4%). In multivariable analysis, low-density CAC volume was independently predictive of MACE (log-transformed; CAC group: HR: 1.65; 95% CI: 1.05-2.60; SPECT group: HR: 1.41, 95% CI: 1.02-1.94; PET group: HR: 1.34, 95% CI: 1.11-1.61; *P* < 0.05), whereas intermediate and high-density volumes were not (*P* > 0.05). Density CAC volumes improved discrimination and reclassification among all 3 groups (CAC, SPECT, and PET groups: global chi-square improvement: 9.1, 16.1, and 16.6, respectively, *P* < 0.01; net reclassification index: 47.3, [95% CI: 33.2-61.4], 49.6 [95% CI: 36.8-62.4] and 15.4, [95% CI: 5.9-24.9], respectively, *P* < 0.01).

**Conclusions:**

Low-density HU volume was independently associated with an increased MACE risk and improved discrimination and reclassification over conventional approaches in a broad spectrum of individuals undergoing CAC scanning.

Coronary artery calcium (CAC) is a marker of coronary atherosclerosis that provides strong predictive value for future adverse outcomes and guides preventive management.^1^ The CAC Agatston score is the most widely used tool for quantifying CAC burden, based on plaque area and plaque-specific density factors, with a higher weighting factor applied to densely calcified plaques.^2^ Previous large-scale studies have shown the strong association between CAC Agatston score and future cardiovascular disease events.^3^, ^4^, ^5^

Emerging evidence suggests that plaque Hounsfield unit (HU) density in computed tomography (CT) imaging reflects the stage of coronary atherosclerosis. In a study using noncontrast CAC scans, low-density CAC transformed into high-density CAC, facilitated by cholesterol management, including statins.^6^^,^^7^ This high-density calcified plaque was associated with a lower cardiovascular disease risk compared to low-density calcified plaque.^8^ Similar findings have been observed in coronary CT angiography (CCTA) studies, where low-density noncalcified plaque showed a stronger association with adverse cardiovascular outcomes than higher density calcified plaque.^9^^,^^10^ Statin use has been shown to decrease low-density noncalcified plaque and increase the overall density of calcified plaque, suggesting a stabilization process.^11^^,^^12^

Recent advances in deep learning (DL) now allow for the automated evaluation of CAC with high accuracy and reproducibility.^13^^,^^14^ We hypothesize that a quantitative approach of density-based CAC volume provides additional prognostic insights over conventional CAC measures derived by DL, positing that a high volume of low-density CAC may represent a large burden of unstable plaque, potentially associated with the future risk of major adverse cardiac events (MACEs). This approach may improve the prediction of adverse outcomes compared to the standard Agatston score.

To test this hypothesis, we explored the relationship between CAC subtype volumes at predefined densities, as assessed by CAC volume (mm^3^) according to CAC HU and MACE in a diverse patient cohort. This cohort included primarily low-risk, asymptomatic patients undergoing standalone CAC scanning for risk assessment, as well as higher-risk patients undergoing concomitant CAC scanning during either single-photon emission CT (SPECT) or positron emission tomography (PET) myocardial perfusion imaging (MPI), used for the diagnostic work-up of suspected coronary artery disease (CAD).

## Methods

### Study population

We evaluated a total of 7,176 patients who underwent CAC scanning with no prior CAD history: 2,028 asymptomatic patients who underwent standalone CAC scanning (CAC group), 2,782 who underwent SPECT-MPI (SPECT group), and 2,366 who underwent PET-MPI (PET group). The CAC group was comprised of asymptomatic subjects enrolled in the EISNER (Early Identification of Subclinical Atherosclerosis by Noninvasive Imaging Research) registry who completed long-term 14-year prognostic follow-up.^15^ The EISNER registry included adult asymptomatic subjects aged between 45 to 80 years who underwent CAC scanning between September 1998 and May 2005. The SPECT group included patients without known CAD who underwent SPECT MPI with gated noncontrast CT scanning at Saint Lukes Health System in Kansas City, Missouri, between 2010 and 2017. The PET group was a cohort of 2,366 patients who underwent rubidium-82 PET MPI with noncontrast CT scanning at Cedars-Sinai Medical Center between 2010 and 2018.^16^

Informed consent for research participation was collected at each medical center. Approval for the conduct of this study and follow-up for all imaging tests at Cedars-Sinai Medical Center was approved by the Cedars-Sinai Medical Center Institutional Review Board.

### Clinical variables

Baseline information including demographics and CAD risk factors were collected using history taking and a medical questionnaire. Hypercholesterolemia was defined as either a pre-existing diagnosis of high cholesterol or ongoing treatment with lipid-lowering agents. Hypertension was noted if patients had a prior diagnosis of hypertension or were under treatment with antihypertensive medications. Smoking status was determined by self-reported current smoking. Diabetes was identified based on a prior diagnosis or current use of diabetes medications. A family history of premature CAD was recorded if a primary relative had been diagnosed with CAD or experienced a cardiac event before the age of 55 for males and 65 for females. Statin use information was collected at the time of the imaging tests, but was not available in the SPECT cohort.

### Image acquisition

All patients underwent imaging testing in accordance with guidelines.^17^, ^18^, ^19^

For the CAC cohort, all subjects underwent baseline noncontrast CAC scanning CT on an electron beam CT scanner (e-Speed, GE Healthcare) or a 4-slice CT scanner (Somatom Volume zoom, Siemens Medical Solutions). The electrocardiogram (ECG)-gated slices were obtained during a single breath hold, with a tube voltage of 120 kVp and reconstructed slice thickness of 2.0, 2.5, or 3 mm.

For the SPECT cohort, all scans were performed using SPECT/CT scanner (Siemens Symbia scanner). Exercise or pharmacological stress was performed per the standard guidelines.^17^ During the SPECT-MPI scanning, noncontrast ECG-gated CTs were obtained, with a tube voltage of 120 to 130 kVp, current 80 mAs, pixel size 0.25 mm to 0.5 mm, and shallow breath protocol. Images were reconstructed with 3 mm slice thickness.

For the PET cohort, all scans were performed using a hybrid Biograph 64 (Siemens Healthcare) PET/CT scanner using pharmacologic stress testing. Before the PET acquisition, ECG-gated noncontrast CT was performed for CAC scoring with a scan protocol of ECG gating, tube voltage of 120 kVp, and tube current of 85 to 150 mA. Images were reconstructed with 3 mm slice thickness.

## CAC analysis

### DL-based CAC segmentation

We used our previously validated DL model for CAC segmentation.^20^^,^^21^ In brief, the model was comprised of two DL networks. The first network was trained for segmentation of the heart silhouette and the second network was trained to segment the CAC. Convolutional long-term short memory models were employed with 3 slices provided to each network as input. The model was trained (n = 2,500) and validated (n = 500) on ECG-gated noncontrast cardiac CT scans.

### Conventional CAC measures

The CAC Agatston score was calculated using methods described by Agatston et al.^2^ by multiplying the calcified plaque area within each CT slice by a weighting factor (1-4) based on peak plaque density. Density score was calculated using methods described by Criqui et al (area score = volume score/appropriate slice thickness; density score = Agatston score/area score).^8^ The density score reflects the average radiographic density of calcified plaque. Higher density scores may indicate heavily calcified plaques, whereas lower density scores may represent less dense calcified plaques. The volume score was calculated as the sum of lesion areas multiplied by slice thickness, a direct measure of the overall burden of CAC without accounting for plaque density. The CAC Agatston score, and total volume were categorized as 0, 1 to 100, 101 to 400, 401 to 1,000, and >1,000.

### Density-based subtype CAC volumes

The total CAC volume was subclassified as low, intermediate, and high-density volume using HU cutoff values in HU (low: 130-199 HU, intermediate: 200-399 HU, and high: ≥400 HU). Each density subtype volume (low density volume, intermediate density volume, and high-density volume) was expressed as a continuous variable in mm^3^. The percent of density volume was calculated as subtype density volume divided by total volume.

### MPI analysis

Quantitative perfusion was assessed by automated quantitation of total perfusion deficit (TPD) (QPS Software, Cedars-Sinai).^22^ The magnitude of myocardial ischemia was quantified by ischemic TPD (stress TPD − rest TPD). For PET-MPI, global myocardial blood flow was calculated at stress and rest with a 1-tissue compartment kinetic model (QPET Software, Cedars-Sinai). Myocardial flow reserve was calculated stress/rest myocardial blood flow.

### Patient outcomes

The primary endpoint for this study was MACE, which encompassed mortality, nonfatal myocardial infarction, unstable angina, and late revascularization, and was evaluated across all 3 patient cohorts. For the CAC cohort, MACE was assessed through clinical visits, phone calls, and mail correspondence. Adverse events were confirmed by 2 independent cardiologists through a review of comprehensive medical, hospital, and death records. In the SPECT cohort, MACE was determined using site-specific methods, including direct patient interviews, email or telephone contact, and review of electronic medical records. Mortality status was verified using the Social Security Death Index, whereas nonfatal myocardial infarction, unstable angina, and late revascularization were adjudicated by an experienced cardiologist. This process was based on symptoms, cardiac enzyme levels, electrocardiographic changes, and imaging modalities such as echocardiography, stress testing, and coronary angiography via CT or catheterization. For the PET cohort, all-cause mortality was determined using the National Death Index and electronic medical records. Other events were identified using electronic medical records, with all events adjudicated according to the standard criteria.

### Statistical analysis

Continuous variables are presented as mean ± SD, and categorical variables as counts (proportions). Continuous variables were compared using the Student's *t*-test or rank-sum test for 2 groups, or 1-way analysis of variance or Kruskal–Wallis test for more than 2 groups. Categorical variables were compared using the Pearson chi-square test. Correlation between imaging metrics was assessed using Pearson correlation, with R-values displayed in heatmap plots. Corresponding 95% CIs were calculated using Fisher z transformation. Cox proportional hazards regression was used to calculate HRs with 95% CIs. The proportional hazards assumption was evaluated using Schoenfeld residual-based tests. Variables that violated this assumption were included as interactions with a function of time to form time-varying covariates. Multivariable analysis was performed after adjusting for age, sex, body mass index, hypertension, diabetes, hyperlipidemia, smoking, family history of premature CAD, ischemic TPD for SPECT/PET, and myocardial flow reserve. The CAC Agatston score, volume, and density volumes were used as log transformed form. Global chi-square analysis was used to evaluate the incremental discriminative value of adding low-density CAC volume to the baseline model, which included age, sex, hypertension, diabetes, hyperlipidemia, smoking status, family history of premature CAD, ischemic TPD in both SPECT and PET groups, and myocardial flow reserve in the PET group only. The area under the receiver operating characteristic curve (AUC) was calculated to assess model discrimination, and comparisons between models were performed using the DeLong test. Model calibration and fit were evaluated using the Akaike information criterion and Bayesian information criterion, with lower values indicating better model performance. Decision curve analysis was conducted to assess clinical utility by quantifying the net benefit of each model across a range of threshold probabilities for predicting MACE. Continuous net reclassification index was used to evaluate the added value of density-based CAC volume information in risk reclassification. All data were analyzed using STATA (version 18; StataCorp LP).

## Results

### Baseline characteristics

The clinical characteristics of the 3 patient groups are summarized in Table 1. The mean age, as well as the prevalence of hypertension and diabetes, were lowest in the CAC group and highest in the PET group. The CAC group had a higher proportion of males compared to the other 2 groups. Body mass index and smoking prevalence were higher in the SPECT group compared to the CAC and PET groups. The frequency of hypercholesterolemia was highest in the CAC group, followed by the SPECT and PET groups. The annualized rate of MACE was significantly higher in PET and SPECT group (4.9% [95% CI: 4.5%-5.4%] and 4.2% [95% CI: 3.8%-4.9%], respectively) and low in the CAC group (0.8% [95% CI: 0.6%-0.9%]).

### Conventional CAC measures

Table 1 shows the CAC characteristics across the 3 groups. CAC Agatston scores and volumes were highest in the PET group, followed by the SPECT and CAC groups. The distribution of patients across CAC categories indicated a higher proportion of zero CAC scores in the CAC group, whereas the PET group had a larger proportion of patients with scores >1,000. Density scores also varied significantly, with the CAC group having the highest density score, followed by the SPECT and PET groups.

### Density-based subtype CAC volumes

The PET group exhibited the highest of all 3 density subtype volumes, followed by SPECT and CAC group. Regarding the proportion of each density volume, the SPECT cohort showed the highest proportion of low-density volume and lowest proportion of intermediate density volume (Table 1). The proportion of high-density volumes were relatively similar across all cohorts.

**Table 1:** Baseline Characteristics

Variables	CAC Group (n = 2,028)	SPECT Group (n = 2,782)	PET Group (n = 2,366)	*P* Value for Differences Between 3 Groups
Age (y)	55.6 ± 9.1	61.7 ± 11.6	69.5 ± 12.2	<0.001
Male	1,196 (59.0)	1,279 (46.0)	1,165 (49.2)	<0.001
BMI (kg/m^2^)	26.6 ± 4.9	33.1 ± 8.9	28.7 ± 7.1	<0.001
Hypertension	818 (40.3)	1,944 (69.9)	1,744 (73.7)	<0.001
Hypercholesterolemia	1,406 (69.3)	1,784 (64.1)	1,413 (59.7)	<0.001
Diabetes	114 (5.6)	731 (26.3)	719 (30.4)	<0.001
Smoking	125 (6.2)	359 (12.9)	188 (8.0)	<0.001
Family history of premature CAD	617 (30.4)	1,201 (43.2)	374 (15.8)	<0.001
Statin use	434 (21.5)	NA^a^	1,224 (51.8)	
MACE rate	216 (10.7)	260 (9.4)	557 (23.6)	<0.001
Follow-up duration (y)	14.4 (13.4-15.8)	2.6 (1.8-3.5)	4.4 (2.6-6.6)	<0.001
Person-years	27,998.2	6,125.9	11,245.6	
Annualized rate (%/y, with 95% CI)	0.8 (0.6-0.9)	4.2 (3.8-4.9)	4.9 (4.5-5.4)	<0.001
Conventional CAC metrics				
CAC score	0 (0-53.6)	5.9 (0-104.6)	180.6 (13.1-719.9)	<0.001
CAC volume	0 (0-57.7)	12.6 (0-115.1)	106.2 (10.2-390.2)	<0.001
CAC category				<0.001
0	1,143 (56.4)	1,149 (41.3)	596 (25.2)	
1-100	503 (24.8)	923 (33.2)	600 (25.4)	
101-400	237 (11.7)	372 (13.4)	512 (21.6)	
401-1,000	109 (5.4)	167 (6.0)	336 (14.2)	
>1,000	36 (1.8)	171 (6.2)	322 (13.6)	
Density score	3.14 ± 0.64	2.64 ± 0.99	1.84 ± 0.32	<0.001
Density volume based on HU				
Low density	0 (0-29.5)	8.4 (0-62.7)	55.9 (6.9-188.8)	<0.001
Intermediate density	0 (0-22.3)	0 (0-40.2)	1.3 (40.1-158.8)	<0.001
High density	0 (0-2.3)	0 (0-25.4)	4.0 (0-40.5)	<0.001
% Low density	47.9 (39.1-62.4)	55.1 (40.3-84.6)	40.1 (32.7-44.3)	<0.001
% Intermediate density	40.8 (33.3-46.0)	37.1 (15.4-44.4)	51.3 (41.7-64.0)	<0.001
% High density	6.4 (0-15.4)	4.0 (0-17.5)	6.3 (0.3-13.4)	0.005

Values are mean ± SDs, n (%), or median (IQR).

BMI = body mass index; CAC = coronary artery calcium; CAD = coronary artery disease; HU = Hounsfield units; MACE = major adverse cardiovascular event; PET = positron emission tomography; SPECT = single-photon emission computed tomography.

a No statin information in SPECT group.

**Figure 1:**
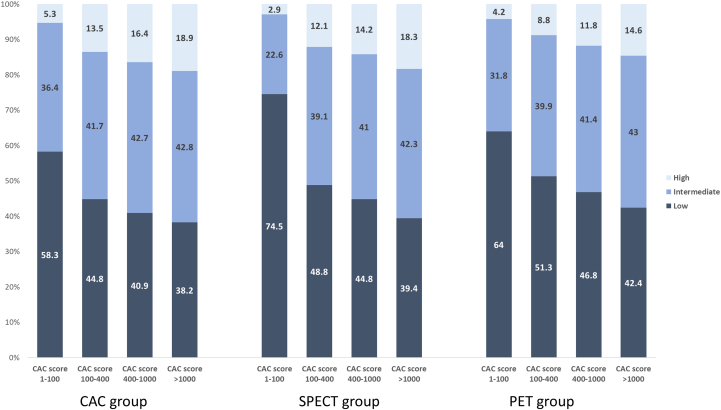
**Distribution of Coronary Artery Calcium Density Volumes According to Coronary Artery Calcium Score Category** Proportions of CAC density volumes based on Hounsfield unit thresholds are shown for each CAC score group (1-100, 101-400, 401-1,000, and >1,000) across the CAC, SPECT, and PET cohorts. Numbers within bars indicate percentages. CAC = coronary artery calcium; SPECT = single-photon emission computed tomography; PET = positron emission tomography.

Figure 1 illustrates the distribution of CAC density volumes according to CAC Agatston score categories. Low-density CAC volume was most prevalent in the lower Agatston score categories, whereas intermediate-density CAC volume increased as Agatston scores rose. High-density CAC volume was minimal in the lower Agatston score categories (ie, 1-100 or 101-400) but became more prominent in higher categories, particularly in the >1,000 category.

**Figure 2:**
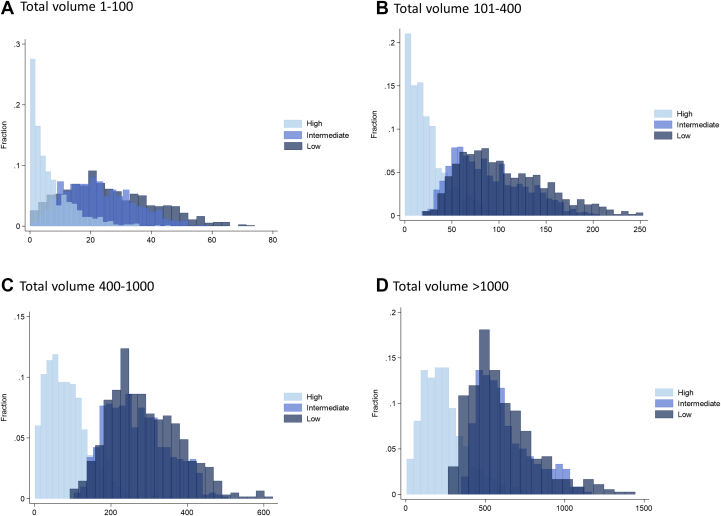
**Histograms for Coronary Artery Calcium Density Volume According to Total coronary Artery Calcium Volume** Distributions of low-, intermediate-, and high-density CAC volumes are shown across total CAC volume categories, (A) 1-100, (B) 101-400, (C) 401-1,000, and (D) >1,000, for the combined study population (all 3 cohorts).

Figures 2A to 2D shows histograms of CAC density volumes across the study population, categorized by total CAC volume ranges (1-100, 101-400, 401-1,000, and >1,000). In patients with low total CAC volumes, low- and intermediate-density CAC volumes were more prevalent, whereas high-density CAC volume was relatively small. As the total CAC volume increased, the proportion of low- and intermediate-density CAC decreased, whereas the proportion of high-density CAC volume increased.

Supplemental Figure 1 shows a heatmap of correlations between CAC parameters, including CAC Agatston score, total volume, density score, and stratified low-, intermediate-, and high-density volumes. The CAC Agatston score and total CAC volume exhibited strong correlations with low-, intermediate-, and high-density volumes (low-density volume: r = 0.907 [95% CI: 0.904-0.912] and 0.971 [95% CI: 0.967-0.975]; intermediate-density volume: r = 0.947 [95% CI: 0.944-950] and 0.951 [95% CI: 0.948-0.953]; high-density volume: r = 0.862 [95% CI: 0.856-0.868] and 0.853 [95% CI: 0.848-0.857], respectively). The density score had moderate correlations with intermediate (r = 0.448 [95% CI: 0.421-0.475]) and high-density volumes (r = 0.407 [95% CI: 0.379-0.435]), but weaker with low-density volume (r = 0.207 [95% CI: 0.179-0.234]).

### Density volume and MACE risk

Unadjusted analysis indicated all CAC density volumes were associated with an increased risk of MACE across all 3 cohorts (Supplemental Table 1) (all *P* < 0.05). Table 2 presents the results of multivariate Cox regression analyses for MACE risk. The proportional hazards assumption was assessed for all 3 cohorts. The CAC group met the proportional hazards assumption, and no adaptations to the model were necessary. However, the proportional hazards assumption was not met for the SPECT and PET groups. Therefore, time-varying covariates were incorporated into the models for these groups to appropriately address violations of the assumption. After adjustment for clinical and imaging factors, only low-density CAC volume was independently associated with an increased risk of MACE (CAC group: HR: 1.65, 95% CI: 1.05-2.60; SPECT group: HR: 1.41, 95% CI: 1.03-1.92; PET group: HR: 1.34, 95% CI: 1.12-1.60; all *P* < 0.05), while intermediate and high-density volumes were not associated with MACE risk (all *P* > 0.05).

**Table 2:** Multivariable Cox Regression Analysis for MACE Risk Including Time-Varying Covariate Adjustment

Variables	CAC Group		SPECT Group		PET Group	
	HR (95% CI)	*P* Value	HR (95% CI)	*P* Value	HR (95% CI)	*P* Value
Age	1.02 (0.99-1.03)	0.080	0.99 (0.97-1.00)	0.099	1.01 (1.00-1.02)	0.152
Male	1.30 (0.96-1.76)	0.091	1.05 (0.81-1.38)	0.694	1.11 (0.93-1.32)	0.262
BMI	**1.04 (1.01-1.06)**	**0.006**	1.00 (0.98-1.02)	0.976	0.97 (0.95-0.98)	**<0.001**
Hypertension	**1.56 (1.17-2.08)**	**0.003**	1.08 (0.77-1.52)	0.641	1.14 (0.92-1.42)	0.221
Diabetes	1.13 (0.70-1.83)	0.624	1.40 (1.07-1.83)	**0.015**	1.14 (0.95-1.38)	0.158
Hypercholesterolemia	0.99 (0.71-1.38)	0.974	0.83 (0.62-1.10)	0.187	0.83 (0.68-1.02)	0.073
Smoking	0.95 (0.55-1.64)	0.843	1.19 (0.83-1.70)	0.345	1.07 (0.75-1.51)	0.714
Family history of premature CAD	1.33 (0.99-1.78)	0.052	0.81 (0.63-1.05)	0.112	0.95 (0.58-1.54)	0.832
Statin use	0.87 (0.63-1.22)	0.427	NA		0.83 (0.62-1.11)	0.213
Ischemic TPD (per %)	NA		1.13 (1.10-1.15)	**<0.001**	1.05 (1.02-1.07)	**<0.001**
MFR (per unit)	NA		NA		0.68 (0.60-0.77)	**<0.001**
CAC Agatston score	0.64 (0.32-1.30)	0.221	0.86 (0.43-1.70)	0.662	0.94 (0.88-1.00)	0.056
CAC density volume						
Low density	1.65 (1.05-2.60)	**0.029**	1.41 (1.03-1.92)	**0.043**	1.34 (1.12-1.60)	**0.001**
Intermediate density	1.35 (0.78-2.35)	0.286	1.20 (0.73-1.95)	0.473	0.88 (0.71-1.08)	0.225
High density	1.15 (0.97-1.37)	0.104	1.08 (0.93-1.25)	0.330	1.10 (0.99-1.21)	0.077
Time-varying covariates adjustments						
Age	NA		1.00 (1.00-1.01)	0.388	1.00 (1.00-1.01)	0.062
Ischemic TPD (per %)	NA		0.95 (0.92-0.97)	**<0.001**	NA	
Family history of premature CAD	NA		NA		0.83 (0.69-0.99)	**0.043**
Statin use	NA		NA		1.06 (0.98-1.14)	0.150

NA indicates not applicable or data unavailable.

Significance *P* values are in **bold**.

MFR = myocardial flow reserve; TPD = total perfusion deficit; other abbreviations as in Table 1.

**Figure 3:**
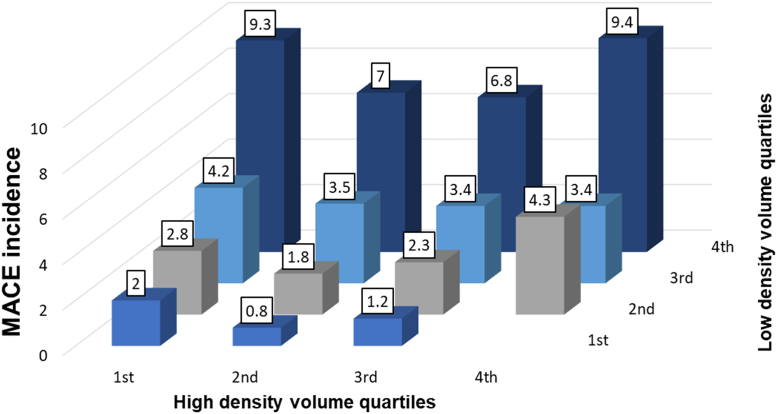
**Major Adverse Cardiovascular Event Incidence According to Low-Density and High-Density Quartiles** MACE incidence stratified by low and high density CAC volume quartiles. MACE = major adverse cardiovascular event.

Figure 3 shows MACE incidence stratified by low- and high-density CAC volumes. MACE incidence increased with higher low-density CAC volumes within each high-density volume quartile (all *P* value <0.01). However, increase in high-density volume were not associated with increased MACE risk (all *P* value >0.05 across high-density volume quartiles.

Table 3 shows the global chi-square analysis for discrimination and net reclassification index analysis for risk reclassification for MACE. The addition of low-density CAC volume as a variable over risk factors and imaging findings significantly improved MACE prediction and reclassification (For CAC, SPECT, and PET groups, global chi-square improvement: 9.1, 16.1 and 16.6, respectively, all *P* < 0.01; net reclassification index: overall 47.3 (95% CI: 33.2-61.4), 49.6 (95% CI: 36.8-62.4) and 15.4 (95% CI: 5.9-24.9), respectively (all *P* < 0.01).

**Table 3:** Global Chi-Square and NRI Analysis

Group	Chi-Square Value				NRI (95% CI)			
	Baseline	Baseline ± Low-Density Volume	Difference	*P* Value	Overall	Case	Noncase	*P* Value
CAC group	213.6	222.7	9.1	0.003	47.3 (33.2-61.4)	17.2 (3.1-31.3)	30.1 (16.0-44.2)	<0.001
SPECT group	263.0	279.1	16.1	<0.001	49.6 (36.8-62.4)	29.7 (16.9-42.5)	20.9 (8.1-33.7)	<0.001
PET group	288.9	305.5	16.6	<0.001	15.4 (5.9-24.9)	19.6 (10.1-29.1)	−4.2 (−13.7 to 5.3)	0.002

NRI = net reclassification index; other abbreviations as in Table 1.

**Figure 4:**
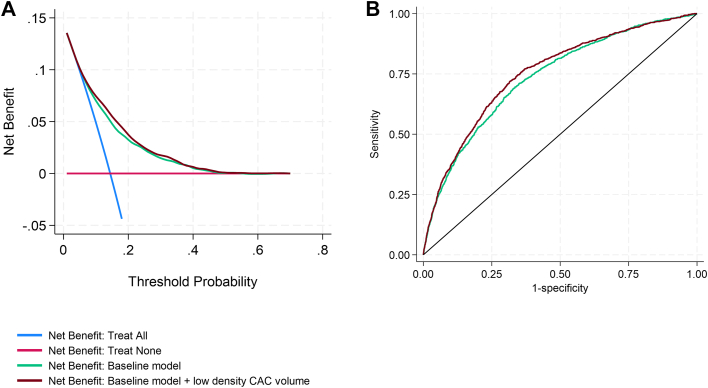
**Decision Curve and Receiver Operating Characteristic Curve Analysis for Major Adverse Cardiovascular Event Prediction** (A) Decision curve analysis (DCA) demonstrates the additive clinical benefit of low-density CAC volume, primarily for individuals at intermediate risk of MACE. (B) ROC analysis indicates that adding low-density CAC volume to the baseline model improved its discriminatory ability (increased AUC, *P* < 0.001). Abbreviations as in Figure 1.

Figure 4A shows decision curve analysis for MACE prediction. Addition of low-density CAC volume to the baseline model resulted in improved net clinical benefit, particularly among patients with low to intermediate predicted probabilities of MACE. Figure 4B presents the AUC analysis, demonstrating a significant increase in discrimination when low-density CAC volume was included in the model (AUC 0.753 vs 0.735, *P* < 0.001). Furthermore, Inclusion of low-density CAC volume in the model substantially improved model fit, with the Akaike information criterion decreasing from 5,280.1 in the baseline model to 5,181.1, and the Bayesian information criterion decreasing from 5,348.9 to 5,277.4. These reductions indicate better overall model performance with the addition of low-density CAC volume.

Supplemental Figure 2 summarizes the workflow of using DL for the analysis and classification of CAC. While conventional methods could quantify the total CAC burden, the DL approach provided for a more detailed assessment by classifying calcium types based on their density. This approach identified coronary plaques with predominantly high-density CAC (light blue arrows), intermediate-density CAC (blue arrows), and low-density CAC (dark blue arrows) and quantified the volume of CAC at varying density levels on a per-patient or per-lesion basis.

## Discussion

In this study, we explored the artificial intelligence-derived fully automated quantitative CAC volumes based on HU density and their association with the future MACE risk among a diverse cardiac imaging population who underwent CAC scanning or ECG-gated noncontrast CT scan as part of cardiac imaging tests (Central Illustration). We found that low-density CAC volume, defined as CAC volume with HU ranging 130-199, was an independent predictor of future MACE and provided additional discrimination when added to conventional approaches, including risk factors, CAC Agatston score, and other cardiac imaging findings across all 3 groups. The current study demonstrates that a fully quantitative approach to calcified plaque volume subtype characterization is feasible, and the subtype volumetric approach of CAC may enhance the risk stratification among various patient populations undergoing cardiac imaging tests.

**Central Illustration:**
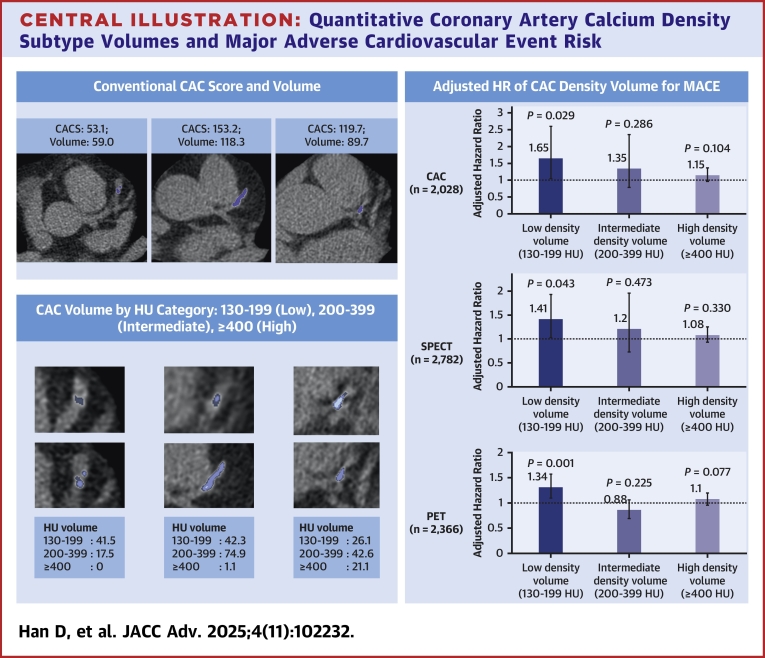
**Quantitative Coronary Artery Calcium Density Subtype Volumes and Major Adverse Cardiovascular Event Risk** Left-side panels compare conventional CAC scoring (upper left) with density-based CAC volume analysis by HU category (lower left), illustrating variability in plaque composition—from predominantly low-density to high-density calcified plaques. The right-side panel summarizes the risk of MACE associated with each CAC density subtype across imaging cohorts. CAC = coronary artery calcium; CACS = CAC Agatston score; HU = Hounsfield units; MACE = major adverse cardiovascular event; SPECT = single-photon emission computed tomography; PET = positron emission tomography.

Our study showed that low-density CAC volume was independently associated with an increased MACE risk, whereas high- or intermediate-density CAC volume were not when accounting clinical risk factors and CAC score. These findings align with previous studies in plaque imaging by CT, where low-density CAC is more closely associated with adverse cardiovascular events compared to those with higher density. Criqui et al demonstrated that on a per-patient basis, a low-density score from CAC scanning was associated with a significantly higher risk of all-cause and cardiovascular disease mortality compared to patients with a high-density score.^8^ Studies with CCTA also showed that the prognostic significance of plaque volume is varied according to its HU subtypes. In a matched case-cohort study, patients with acute coronary syndrome had a higher volume of low-density noncalcified plaque and significantly lower high-density calcified plaque compared to patients without acute coronary syndrome.^9^ In addition, in a study from the SCOT-HEART (The Scottish Computed Tomography of the Heart) trial, low-density noncalcified plaque was found to be an independent predictor of MACE risk, whereas calcified plaque volume was not.^10^

Previous histopathology or coronary imaging studies have shown the natural progression of plaque in coronary atherosclerosis involving changes in plaque density. The initial development of coronary plaque involves macrophages and smooth muscle cells within noncalcified plaque and necrotic core.^23^^,^^24^ As plaque progresses, these intraplaque components release free calcium and phosphorus, leading to microcalcification and eventually large, dense calcifications.^25^, ^26^, ^27^ This progression can be influenced by cholesterol-lowering medications, such as statin, ezetimibe, and proprotein convertase subtilisin/kexin type 9 inhibitors.^28^, ^29^, ^30^ Serial CAC studies have shown that the statin use accelerated CAC score progression.^31^^,^^32^ CCTA studies further provide insights into specific plaque subtypes, showing a decrease in low-density plaque and an increase in overall and high-density calcified plaque volume with statin treatment.^12^ These prior findings suggest that density of calcified plaque may reflect different stages in the calcification cascade.

In the current study, we thoroughly assessed the associations among CAC parameters, including the conventional CAC Agatston score, volume, and the new density-based CAC volumes. Although low- and intermediate-density CAC contributed substantially to total CAC volumes, their relative contribution decreased as the total CAC volume increased (Figures 2A to 2D). Conversely, the proportion of high-density CAC volume became more prominent with increasing CAC burden, indicating a shift in plaque composition toward more densely calcified plaques in patients with higher CAC burdens. This shift reflects the evolution of CAC from low-density calcified plaques to high-density calcified plaques, aligning with previous findings from prior CAC and CCTA studies. Therefore, our study findings suggest that the quantitative density-based volumetric approach for CAC subtype could capture the stages of the calcification process using noncontrast CT imaging. The higher low-density CAC volume may represent an earlier stage of calcification, which poses a higher risk compared to later-stage, more dense calcified plaques.

Recent findings from the MESA (Multiethnic Study of Atherosclerosis) study align with the current study in highlighting the prognostic relevance of CAC density.^33^ That study demonstrated that higher CAC density was associated with a lower coronary heart disease risk at lower CAC volumes (≤130 mm^3^), and that evaluating density and volume separately improved risk prediction. However, the use of 2 independent parameters—volume and density—may limit clinical applicability due to interpretative complexity. In contrast, our study integrates both plaque density and volume into a single, fully quantitative metric by measuring the absolute volume of low-density CAC subtypes, which was independently associated with the MACE risk. This approach simplifies clinical interpretation, potentially offering a practical and automated tool for risk stratification.

We validated the new quantitative CAC subtype volume approach not only in asymptomatic patients with CAC stand-alone scanning but also in patients who underwent nuclear imaging tests that often include CAC scanning as part of the studies. Our findings demonstrated a consistent independent relationship between low-density CAC volume and MACE risk in both the SPECT and PET groups. This suggests that this approach may be broadly applicable to a large population undergoing noncontrast ECG-gated CT scanning. Recent studies have shown that the CAC Agatston score in CT attenuation scanning can reliably assess the presence and severity of coronary plaque burden and that these findings are independently associated with the future risk.^14^^,^^16^ Further studies are needed to explore whether the current quantitative method is applicable to CT attenuation scanning or non-ECG-gated noncontrast CT scanning.

The current study provides several insights and potential future applications. The independent prognostic significance of low-density HU volume in predicting MACE suggesting this new imaging marker could guide the intensity of preventive management beyond the conventional CAC category with the Agatston score. Moreover, serial assessment of density-based plaque volume may offer improved risk stratification for patients undergoing statin treatment. Although CAC Agatston scores are well-established predictors of future adverse cardiovascular disease risk, the traditional scoring method may lead to cardiovascular disease risk misclassification, particularly in individuals with highly dense plaques at baseline and on statin therapy.^7^ Serial CAC scanning with measurement of the Agatston CAC score has not proven effective in monitoring therapy due to the well described effect of statins on accelerating coronary calcification, considered to be a process of plaque stabilization.^31^^,^^32^ Evaluating CAC subtype volumes based on density could potentially enhance patient stratification for the preventive treatment. For instance, serial CAC assessments using this method may help identify individuals who exhibit a predominantly increased low-density CAC volume in follow-up scans, indicating a need for more intensive preventive management to stabilize coronary plaque, from low-density CAC to high-density CAC.

### Study Limitations

The current study has several limitations. Due to the observed nature of the current study, we cannot discount the possibility of unmeasured confounding factors that might affect the clinical endpoints of this study. The information regarding downstream pharmacologic management after testing was unavailable. We did not test the correlation between our CAC density-based approach and other plaque imaging modalities, such as CCTA. Further validation is needed to explore the correlation between CAC density measures and CCTA or invasive coronary imaging-defined plaque subtypes. However, given that CAC and CCTA use the similar CT technique, we anticipate that there will be a significant correlation between CAC-defined density and CCTA-defined calcified plaque density. Nevertheless, differentiating low-density calcium from contrast-enhanced components on CCTA can be challenging due to overlapping HU ranges. Emerging technologies such as photon-counting CT and dual-energy CT may enhance plaque characterization by allowing better differentiation of low-density calcium from contrast material.

## Conclusions

A fully automated DL-based method for quantifying CAC volumes according to CT densities is feasible and provides enhanced subclassification of calcified plaque in noncontrast CAC scanning. Low-density HU volume was independently associated with an increased MACE risk, offering additional prognostic insights for a broad spectrum of individuals undergoing CAC scanning.

Perspectives **COMPETENCY IN MEDICAL KNOWLEDGE:** The current study demonstrates that low-density CAC volume—defined by attenuation between 130 to 199 HUs—is independently associated with MACE, beyond conventional risk factors and Agatston score–based stratification. These findings align with prior reports highlighting the added value of CAC density in enhancing the cardiovascular disease risk assessment.

**TRANSLATIONAL OUTLOOK:** Future studies should investigate whether changes in low-density CAC volume over time reflect therapeutic efficacy and risk modulation, potentially offering a noninvasive tool to monitor response to statin or other atherosclerosis-targeted therapies.

## Funding support and author disclosures

This research was supported in part by grant R35HL161195 from the 10.13039/100000050National Heart, Lung, and Blood Institute/10.13039/100000002National Institutes of Health (NHLBI/NIH) (PI: Piotr Slomka) and a grant from the Dr Miriam and Sheldon G. Adelson Medical Research Foundation. Dr Miller has received consulting fees from Pfizer and research support from Pfizer and Alberta Innovates. Drs Berman and Slomka participate in software royalties for QPS software at Cedars-Sinai Medical Center. Dr Slomka has received research grant support from Siemens Medical Systems and consulting fees from Synektik. Dr Berman has received consulting fees from GE Healthcare. All other authors have reported that they have no relationships relevant to the contents of this paper to disclose.
