# Supplementary material for: Association of Quantitative Coronary Artery Calcium Density Subtype Volumes With Major Adverse Cardiovascular Events
Source: JACC Adv. 2025 Oct 23;4(11):102232. doi: 10.1016/j.jacadv.2025.102232 (PMC12593604; doi:10.1016/j.jacadv.2025.102232)
Supplement: Supplemental Data [file mmc1.docx]

**Supplemental Material**

| Supplemental Table 1. Univariate Cox regression analysis for MACE risk | | | | | | |
| --- | --- | --- | --- | --- | --- | --- |
|  | CAC group | | SPECT group | | PET group | |
|  | HR (95% CI) | P-value | HR (95% CI) | P-value | HR (95% CI) | P-value |
| Age | 1.05  (1.04-1.07) | <0.001 | 1.03  (1.01-1.04) | <0.001 | 1.04  (1.03-1.05) | <0.001 |
| Male | 1.56  (1.16-2.08) | 0.003 | 1.94  (1.51-2.49) | <0.001 | 1.08  (0.91-1.29) | 0.373 |
| BMI | 1.05  (1.03-1.07) | <0.001 | 1.00  (0.99-1.01) | 0.957 | 0.95  (0.94-0.97) | <0.001 |
| Hypertension | 2.39  (1.82-3.14) | <0.001 | 1.73  (1.26-2.38) | 0.001 | 1.36  (1.10-1.69) | 0.005 |
| Diabetes | 1.91  (1.20-3.02) | 0.006 | 1.78  (1.39-2.28) | <0.001 | 1.26  (1.04-1.51) | 0.015 |
| Hypercholesterolemia | 1.29  (0.95-1.75) | 0.101 | 1.12  (0.86-1.46) | 0.395 | 0.81  (0.68-0.97) | 0.022 |
| Smoking | 1.04  (0.61-1.79) | 0.877 | 1.34  (0.95-1.89) | 0.091 | 0.76  (0.53-1.09) | 0.139 |
| Family history of premature CAD | 1.30  (0.98-1.72) | 0.066 | 0.84  (0.65-1.08) | 0.167 | 0.49  (0.35-0.68) | <0.001 |
| Statin use | 1.41  (1.04-1.91) | 0.026 | NA |  | 1.01  (0.85-1.21) | 0.886 |
| Ischemic TPD (per %) | NA |  | 1.12  (1.10-1.15) | <0.001 | 1.07  (1.05-1.09) | <0.001 |
| MFR (per unit) | NA |  | NA |  | 0.49  (0.42-0.55) | <0.001 |
| CAC Agatston score | 1.31  (1.28-1.35) | <0.001 | 1.47  (1.39-1.56) | <0.001 | 1.17  (1.13-1.21) | <0.001 |
| CAC density volume | | | | | | |
| Low density | 1.58  (1.48-1.69) | <0.001 | 1.60  (1.49-1.72) | <0.001 | 1.29  (1.23-1.35) | <0.001 |
| Intermediate density | 1.56  (1.46-1.66) | <0.001 | 1.52  (1.43-1.62) | <0.001 | 1.25  (1.20-1.30) | <0.001 |
| High density | 1.57  (1.47-1.68) | <0.001 | 1.46  (1.38-1.54) | <0.001 | 1.23  (1.18-1.29) | <0.001 |
| Abbreviations: CAC, coronary artery calcium; CI, confidence interval; SPECT, Single-photon emission computed tomography; PET, positron emission tomography; BMI, body mass index; CAD coronary artery disease; TPD, total perfusion deficit; MFR, myocardial flow reserve; HR, hazard ratio; MACE, major adverse cardiovascular events | | | | | | |

Supplemental Figure 1. Correlations between CAC parameters in entire study population. Abbreviations: CACS: CAC Agatston score


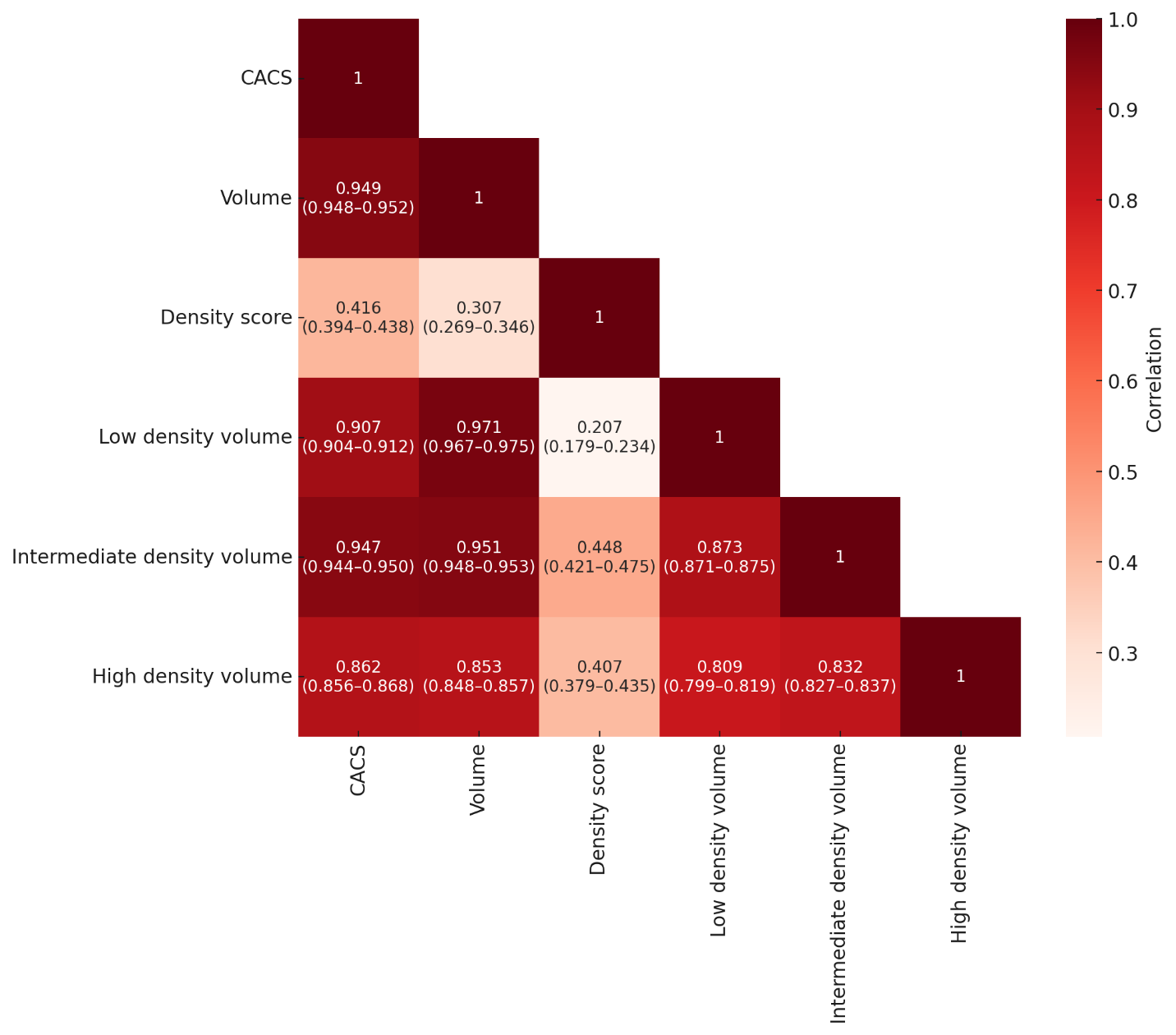


Supplemental Figure 2. Summarization of the workflow of using DL for the analysis of CAC density-based volume.


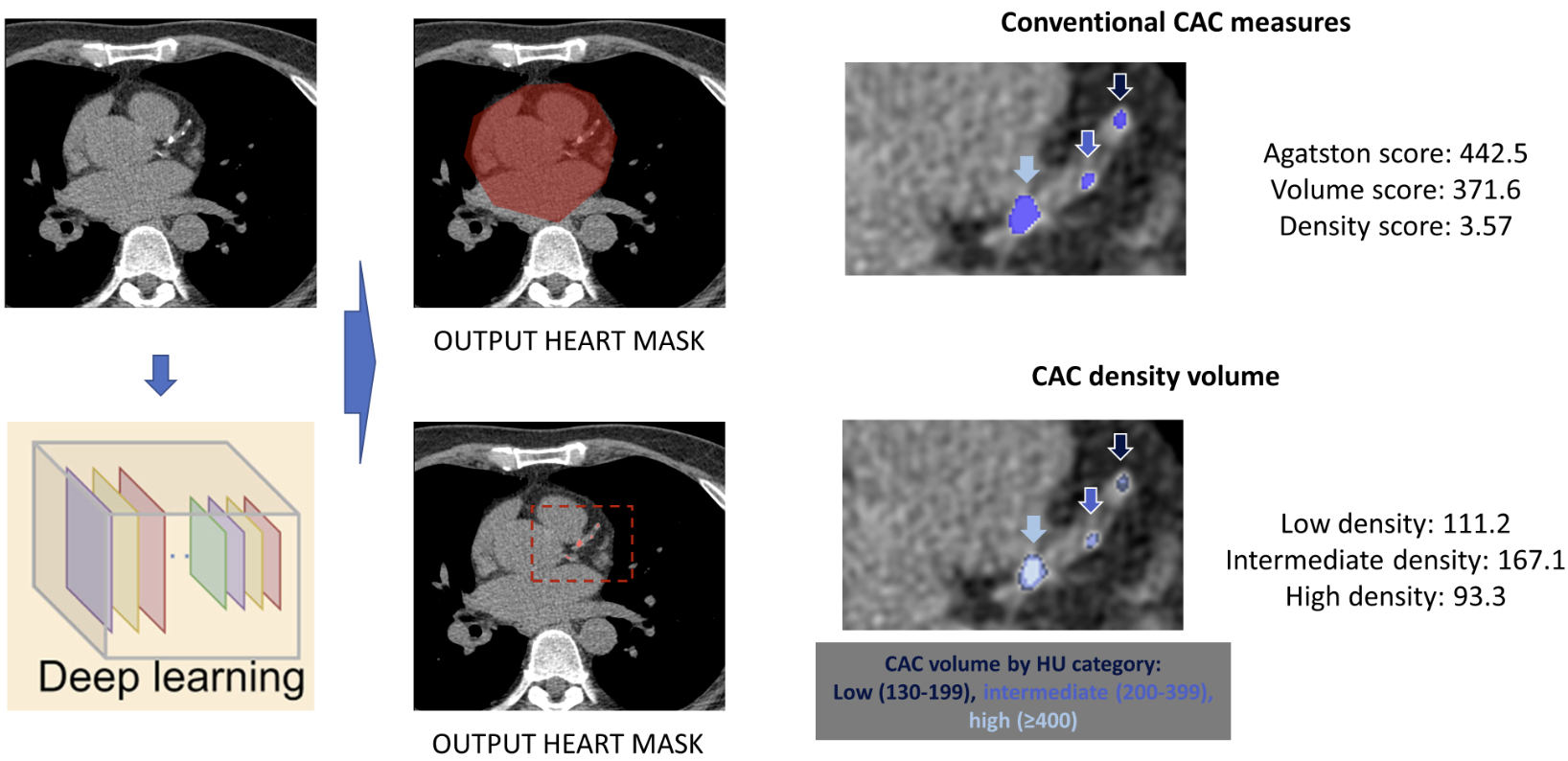


Abbreviations: CAC, coronary artery calcium; HU, Hounsfield Units
